# Spontaneous Gastric Perforation in a Case of Collagenous Gastritis

**Published:** 2016-01-01

**Authors:** Marly H Appelman, Tim G.J de Meij, E. Andra Neefjes-Borst, C.M.F Kneepkens

**Affiliations:** 1 Department of Paediatric Gastroenterology VU University Medical Centre, Amsterdam, The Netherlands; 2 Department of Pathology, VU University Medical Centre, Amsterdam, The Netherlands

**Keywords:** Collagenous gastritis, Gastric perforation, Acute abdomen

## Abstract

Collagenous gastritis is an extremely rare disease, both in children and adults. Symptoms vary depending on the extent of collagenous changes in the bowel. In most of the children, iron deficiency anemia and abdominal pain are the presenting symptoms. We present a 15-year-old boy with acute abdomen due to gastric perforation the cause of which was collagenous gastritis.

## CASE REPORT

A 15-year-old, previously healthy boy without a history of smoking or drug abuse, admitted to the hospital with acute epigastric pain without dyspnea or heartburn. On physical examination the boy was pale with tachycardia (110/min), but normal blood pressure (110/70 mmHg) and normal capillary oxygen saturation in room air (SPO2 99%). Tenderness in the upper abdomen was present. Laboratory investigations showed mild iron deficiency anemia (haemoglobin concentration 7.1 mmol/l, mean cellular volume 65 fl, serum iron 4 mmol/l, serum ferritin 3 g/l) with normal infection parameters. X-ray revealed free air under the right diaphragm. Abdominal ultrasound and CT scan were performed in search of underlying abnormalities, but none could be detected. At laparotomy, gastric perforation was found, which was repaired after refreshing the margins and tissue was sent for histopathology. Histopathology of the fragments showed ulceration without demonstrable Helicobacter pylori; recognizable mucosa was lacking. Subsequent gastro-duodenoscopy showed macroscopically a nodular aspect of the corpus with remarkably coarse gastric folds and pseudo-polyps (Fig. 1). Histological examination of gastric biopsies showed active chronic inflammation with lympho-plasma-cellular infiltration and a thickened sub-epithelial collagenous band, up to 23 μm, consistent with the diagnosis collagenous gastritis; the duodenal mucosa was normal.

**Figure:**
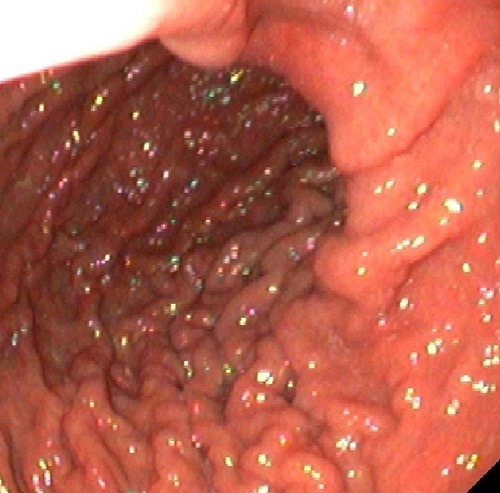
Figure 1:Coarse folds and pseudopolyps in the gastric lumen.

The patient was treated with iron supplementation because of persistent anemia. Repeated gastroscopies over the years yielded similar results with collagen bands of up to 22-29 m (Fig. 2,3); colonoscopy revealed normal colonic mucosa, both macroscopically and on histological examination. At the age of 18 year, the patient is asymptomatic, but needed frequent courses of iron replacement therapy.

**Figure:**
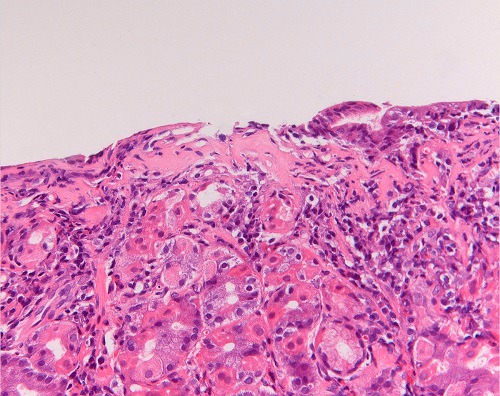
Figure 2:Hypertrophic mucosa with irregularly thickened sub-epithelial membrane. (200x)

**Figure:**
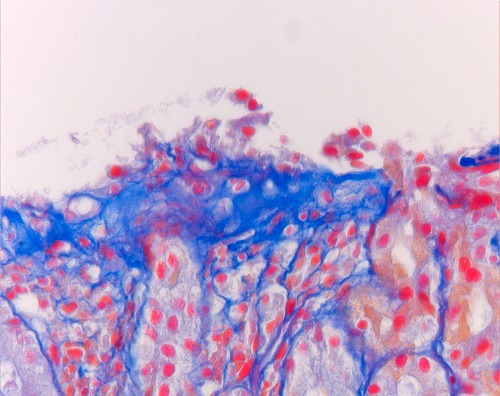
Figure 3:Azan staining of gastric mucosa. Collagen is presented in blue. (400x)

## DISCUSSION

The first report on collagenous gastritis was published in 1989.[1-3] Over 300 patients with collagenous gastritis (40 pediatric) have been reported, the youngest being 9 months old.[4] It is twice as common in females.[5-8]. Collagenous gastritis in adults is often accompanied by collagenous sprue and (or) collagenous colitis; in children and adolescents it is usually an isolated phenomenon.[7] The etiology has not been completely known yet; an autoimmune background is suggested.[4] The presentation is variable in children including upper abdominal pain, anemia, vomiting, upper gastrointestinal bleeding.[4,7-8]. Perforation of the stomach as the presenting symptom of collagenous gastritis is unusual. Leung et al presented a 20-year-old man with perforated gastric ulcers preceding the occurrence of collagenous gastritis by four years.[3] Collagenous gastritis was also reported in literature, in a 15-year-old boy with a bleeding gastric ulcer, but without perforation.[6]

The diagnosis of collagenous gastritis is based on characteristic endoscopic and histological findings. At endoscopy, especially in children and adolescents, the gastric mucosa usually looks macroscopically inflamed, with thickened folds and a nodular or pseudopolypoid as was also seen in our patient.[7] Histologically the sub-epithelial layer is irregularly thickened, not extending around the foveolae and glandular tubes. It consists of collagen; typically, entrapped capillaries are present within this layer and the lamina propria contains an increase in intraepithelial inflammatory cells.[4] The collagen layer is normally is 0.30-2.81 μm (mean, 1.33 μm) and does not seem to change significantly in other forms of chronic gastritis but it is grossly increased to between 15 and 94 μm with a diffuse or patchy distribution in collagenous gastritis. [2,5,9] This phenomenon is pathognomonic; it is found in no other gastrointestinal condition.

There is very limited data on the treatment of collagenous gastritis in children and adults [1,4,7]. Both acid reduction and corticosteroid treatment seem to be mostly ineffective, although many children become symptom-free independent of the type of treatment. Obviously, iron deficiency anemia can successfully be treated with iron supplementation, a treatment that has to be continued for prolonged period. Collagenous gastritis is easily overlooked on account of non-specific features and can lead to gastric perforation. Histological specimens should always be taken during surgical repair, since the diagnosis of collagenous gastritis can only be established on histological findings.

## Footnotes

**Source of Support:** Nil

**Conflict of Interest:** None declared

